# CD4 Count and Routine Laboratory Parameters in Neurological Manifestations of HIV: A Secondary Analysis of Their Diagnostic Utility

**DOI:** 10.7759/cureus.107726

**Published:** 2026-04-26

**Authors:** Neeraj Joshi, Zaw Nyein Aye, Tom Harris, Oluwafemi Akanbi, Harriet Guthrie, Shihab Bappy, Gaurav Joshi, Nirankar Singh Neki

**Affiliations:** 1 Internal Medicine, East Kent Hospitals University NHS Foundation Trust, Margate, GBR; 2 Cardiology, East Kent Hospitals University NHS Foundation Trust, Margate, GBR; 3 Medicine, East Kent Hospitals University NHS Foundation Trust, Queen Elizabeth The Queen Mother Hospital, Margate, GBR; 4 General Practice, University Institute of Pharma Sciences, Chandigarh University, Mohali, IND; 5 Internal Medicine, Punjab Institute of Medical Sciences, Jalandhar, IND

**Keywords:** cd4, cd4 counts, hiv, hiv aids, hiv/aids-related opportunistic infections, hiv neuroinfections

## Abstract

Background

Neurological manifestations are a significant cause of morbidity among individuals living with HIV. In many clinical settings, particularly where access to advanced neuroimaging and cerebrospinal fluid diagnostics is limited, risk stratification often relies on CD4 count and routinely available laboratory parameters. In clinical practice, these parameters are frequently used as surrogate indicators of disease severity; however, their role as triage tools for identifying neurological involvement remains uncertain. This study evaluated whether CD4 count and routine laboratory parameters (haematological, renal, and hepatic) are independently associated with the presence of neurological manifestations in HIV-infected adults. A secondary objective was to assess their ability to distinguish between infectious and non-infectious neurological aetiologies.

Methodology

We performed a hypothesis-driven secondary analysis of a prospective observational cohort study conducted at a tertiary care teaching hospital in India between June 2016 and July 2017. Adults with confirmed HIV infection were evaluated for neurological manifestations based on clinical assessment, neuroimaging, and cerebrospinal fluid findings. CD4 count and routinely available laboratory parameters, including haemoglobin, total leucocyte count, renal and hepatic functions (aspartate and alanine aminotransferase and bilirubin), and serum sodium, were analysed. Multivariable logistic regression adjusted for age, sex, and antiretroviral therapy status was used to assess independent associations. Model discrimination was evaluated using receiver operating characteristic (ROC) curve analysis. Primary and subgroup analyses assessed neurological and infectious versus non-infectious outcomes.

Results

Among 100 adults with HIV infection, neurological manifestations were present in 44 (44%) patients. Of these, 27 (61.4%) were opportunistic central nervous system infections, while 17 (38.6%) were non-infectious neurological diagnoses. Mean CD4 count did not differ significantly between patients with and without neurological involvement (100.27 ± 48.20 vs. 102.00 ± 44.28 cells/µL; p = 0.85). Similarly, routinely measured haematological, renal, and hepatic parameters were not independently associated with neurological manifestations (all p > 0.05). In contrast, on multivariable analysis within the neurological subgroup, lower CD4 count was independently associated with infectious etiology (adjusted odds ratio = 4.30 per 50-cell decrease; 95% confidence interval = 1.18-15.63; p = 0.027). ROC analysis demonstrated poor discrimination for predicting overall neurological involvement (area under the curve (AUC) = 0.60), but moderate discriminative ability for distinguishing infections from non-infectious aetiologies (AUC = 0.73).

Conclusions

CD4 count was not an independent predictor of overall neurological involvement in adults living with HIV, although it remained significantly associated with infectious neurological aetiologies. Therefore, CD4 count reflects the degree of immunosuppression but may not be sufficient as a standalone triage marker for overall neurological involvement. Routine laboratory parameters have limited discriminating value for neurological risk certification when used in isolation. These findings highlight the need for a more integrated clinical approach rather than reliance on single laboratory markers, particularly in resource-constrained settings.

## Introduction

Neurological manifestations remain a major contributor to morbidity and mortality among individuals living with HIV, even in the era of combination antiretroviral therapy (ART) [1-3]. The nervous system may be affected at any stage of infection, with involvement of both the central and peripheral nervous systems presenting as opportunistic infections, neoplasms, cerebrovascular events, myelopathy, peripheral neuropathies, and HIV-associated neurocognitive disorders (HANDs). Despite major improvements in systemic viral suppression, neurological complications persist, reflecting multifactorial pathophysiological mechanisms that extend beyond immunosuppression alone, including chronic neuroinflammation, ongoing central nervous system (CNS) viral persistence, and vascular injury [1-3]. In many low-and middle-income countries, patients still frequently present with advanced immunosuppression and severe neurological disease [2,4,5]. Hospital-based series from India and other resource-constrained settings consistently report a broad spectrum of HIV-related neurological disorders, with opportunistic infections, particularly tuberculous meningitis and cryptococcal meningitis, accounting for a substantial proportion of admissions and adverse outcomes in individuals with low CD4 cell counts. These cohorts highlight the continuing burden of CNS opportunistic infections in settings where ART coverage, diagnostic capacity, and timely access to care remain variable [2,4,5].

At the same time, the neurological phenotype of HIV is evolving. With wider use of ART and improved survival, chronic complications such as HANDs, distal sensory polyneuropathy, and cerebrovascular disease are increasingly recognised, including in individuals with partial immune recovery and virological control [1,3,6,7]. Reviews of HIV-related stroke and broader HIV neurology describe an increased awareness of vascular events, cognitive impairment, and other non-opportunistic neurological complications occurring across a range of CD4 cell counts, driven by a combination of viral, host, and treatment-related factors [1,3,6,7]. This shift has raised important questions about how best to identify patients at risk of neurological involvement in routine clinical practice, beyond simply focusing on profound immunosuppression [1,3,7].

In many resource-limited settings, however, access to advanced neuroimaging, cerebrospinal fluid (CSF) analyses, and specialised biomarkers is restricted [2,4]. As a result, clinicians often rely on readily available laboratory parameters to inform early decision-making. Commonly used tests include CD4 cell count, haemoglobin concentration, total leucocyte count, renal function, liver function tests, and serum electrolytes, which together provide an overview of immune status, co-morbid conditions, and systemic illness burden [2,4,5]. Historically, progressively lower CD4 cell counts have been strongly associated with increased susceptibility to opportunistic infections, including CNS infections, and remain central to risk stratification in many settings [2,4,5]. At the same time, contemporary data show that neurological complications, including stroke and HANDs, can occur at higher CD4 levels in patients on ART, suggesting that CD4 cell count alone may no longer be a reliable marker of neurological involvement [1,3,6,7].

Anaemia is highly prevalent among people living with HIV and has been repeatedly associated with worse clinical outcomes. Large cohort studies and a recent systematic review and meta-analysis indicate that anaemia affects a substantial proportion of adults and children with HIV and that its presence is associated with increased morbidity and mortality [8,9]. Similarly, renal and hepatic dysfunction are common in treated and untreated HIV infection and may reflect chronic viral co-infections, antiretroviral drug toxicity, and ongoing systemic inflammation [10,11]. These routine haematological and biochemical parameters are widely measured and frequently available even in resource-constrained environments. However, the extent to which such parameters, beyond CD4 cell count, independently identify patients who present with neurological manifestations, rather than other severe non-neurological complications, is not well defined in existing literature [2,5].

Most clinical studies of HIV-related neurological disease, particularly from low- and middle-income settings, have focused on describing the spectrum and frequency of neurological syndromes and their association with advanced immunosuppression and CD4 cell count rather than formally assessing the independent predictive value of basic laboratory markers for neurological involvement [2,4,5]. This gap is especially relevant in settings where early triage and diagnostic decisions may depend heavily on these routinely available parameters in the absence of advanced neurodiagnostic tools [2,4].

Given the restricted availability of specialist investigations in many resource-limited settings, we aimed to explore whether commonly available clinical and laboratory parameters (haematological, renal, and hepatic) could serve as practical proxies for assessing the risk of HIV-related neurological disease. Therefore, we conducted a hypothesis-driven secondary analysis of a previously established cohort of individuals with HIV infection [12] to evaluate whether CD4 cell count and routinely available laboratory parameters could serve as practical triage tools for identifying neurological involvement at presentation. Specifically, the primary objective of the study was to assess the utility of CD4 count and routinely available biochemical parameters as independent clinical markers of neurological manifestations at presentation, irrespective of aetiology. The secondary objective was to evaluate the ability of CD4 count and routinely available biochemical parameters to discriminate between infectious and non-infectious neurological aetiologies within affected patients.

We hypothesised that although CD4 count and basic laboratory markers reflect immunosuppression and systemic illness burden, they would have limited utility as standalone triage tools for identifying neurological involvement, but may retain value in differentiating infectious from non-infectious causes.

## Materials and methods

Study design and setting

This study represents a secondary analytic evaluation of data derived from a previously conducted prospective observational cohort of individuals living with HIV infection. The original study was conducted at a tertiary care teaching hospital in North India between June 2016 and July 2017. Patients were consecutively evaluated during the study period. The present analysis was designed to assess whether CD4 count and routinely available laboratory parameters provide independent clinical value in identifying neurological manifestations at presentation, and whether these markers differentiate infectious from non-infectious neurological aetiologies. CD4 counts were measured using standard flow cytometry-based methods available at the tertiary care centre. Routine biochemical parameters were analysed using hospital laboratory protocols, consistent with standard clinical practice during the study period. Specific assay platforms were not recorded in the original data set. Missing data were handled using complete case analysis.

Inclusion and exclusion criteria

The present study is a secondary analysis of a previously conducted prospective cohort. Therefore, the inclusion and exclusion criteria were derived from the original study protocol. All adult patients (>18 years) with confirmed HIV infection presenting with neurological symptoms and/or signs during the study period were included in the analysis. Patients were categorised based on the presence or absence of neurological manifestations at presentation. Patients were recruited from the ART centre and medical wards during the study period. Exclusion criteria in the original cohort included patients with a prior history of neurological disease before HIV diagnosis; a history of diabetes mellitus; a history of substance abuse such as narcotics, sedatives, and hypnotics; and patients with incomplete clinical or laboratory data relevant to the analysis to ensure data integrity.

Study population and case definitions

**Figure 1:**
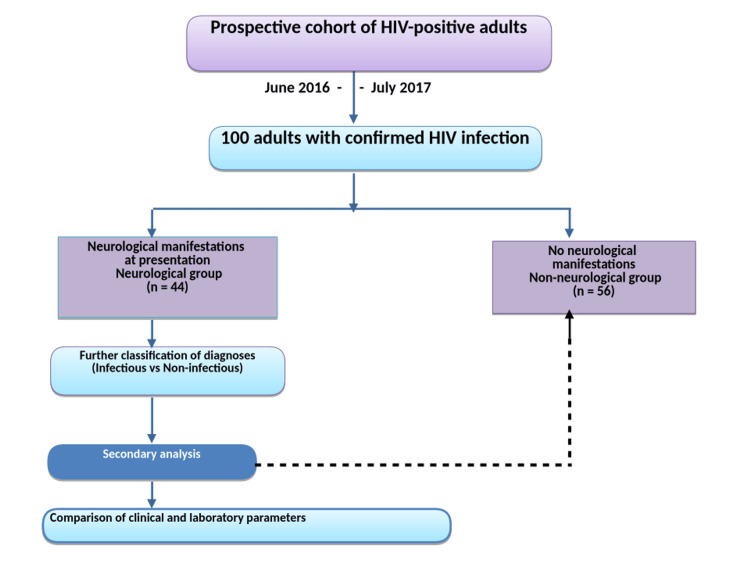
Study design and patient flow of the prospective cohort. This flow diagram illustrates a prospective cohort of 100 adults with confirmed HIV infection and enrolled between June 2016 and July 2017. Participants were categorised into those with neurological manifestations (n = 44) and those without neurological manifestations (n = 56). Neurological diagnoses were further classified into infectious and non-infectious aetiologies. A secondary analysis was performed to compare clinical characteristics and routinely available laboratory parameters, including CD4 count and biochemical indices, between groups.

A total of 100 adult patients with confirmed HIV infection were included (Figure 1). Patients were categorised into the following two groups based on the presence or absence of neurological manifestations at presentation: neurological group: n = 44 (44%), and non-neurological group: n = 56 (56%).

Neurological manifestations were defined based on clinical neurological assessment, supported, where appropriate, by neuroimaging (CT or MRI of the brain and spinal cord) and CSF analysis. Neurological diagnoses were further classified into infectious aetiologies, namely, tubercular meningitis, cryptococcal meningitis, and toxoplasmosis. Tubercular meningitis was diagnosed using CSF parameters, including glucose, protein, and lymphocyte-predominant white cell count, with supportive adenosine deaminase (ADA) levels greater than 10 IU/L. Cryptococcal meningitis was diagnosed using CSF cryptococcal antigen and India ink testing. Toxoplasmosis was diagnosed using *Toxoplasma* IgG serology supported by compatible MRI brain findings. Non-infectious aetiologies included cerebrovascular events, including intracranial haemorrhage, along with other non-infectious neurological conditions. ART status at presentation was recorded.

Variables

Baseline demographic and clinical data were obtained from the original dataset. The following laboratory parameters recorded at presentation were included in the analysis: CD4 count (cells/µL), haemoglobin (g/dL), total leukocyte count (cells/µL), serum creatinine (mg/dL), serum sodium (mmol/L), and liver function parameters including aspartate aminotransferase (AST) (U/L), alanine aminotransferase (ALT) (U/L), and total bilirubin (mg/dL). Age, sex, and ART status were also included as covariates.

Outcome measures

The primary outcome was the presence of neurological manifestations at presentation, based on clinical assessment supported by neuroimaging and/or CSF findings. The secondary outcome was classification of neurological cases into infectious and non-infectious aetiologies within the neurological subgroup.

Statistical analysis

Statistical analyses were conducted using standard statistical software following conventional methods for logistic regression and receiver operating characteristic (ROC) curve analysis. Variables included in the multivariable logistic regression model were selected based on clinical relevance and prior literature, along with findings from univariate analysis, where applicable.

Given the sample size, model complexity was intentionally kept limited to reduce the risk of overfitting, and no interaction terms were included. Standard assumptions for logistic regression were assessed, and results are reported as odds ratios (ORs) with 95% confidence intervals (CIs) and corresponding p-values. Missing data were minimal and handled using complete case analysis.

Continuous variables are presented as mean ± standard deviation (SD) and median (interquartile range (IQR)). Between-group comparisons were performed using appropriate parametric or non-parametric tests according to data distribution. Categorical variables were compared using the chi-square test or Fisher’s exact test, as appropriate.

Multivariable logistic regression was performed for predictors of overall neurological manifestations in the full cohort (n = 100) and predictors of infectious versus non-infectious aetiology within the neurological cohort (n = 44). Variables of clinical relevance and/or those showing statistical significance in univariate analysis were included in the multivariable models.

CD4 count was modelled per 50-cell decrease. In addition, threshold analyses were performed using clinically relevant CD4 cut-offs (<50, <100, and <150 cells/µL).

Model discrimination was assessed using ROC curve analysis with the area under the curve (AUC). A two-sided p-value <0.05 was considered statistically significant.

Ethical considerations

This study represents a secondary analysis of a prospectively collected dataset derived from an institutional research project conducted as part of an MD thesis at Baba Farid University of Health Sciences, Faridkot, India. The original study obtained appropriate institutional ethical approval. The present analysis utilised anonymised, de-identified data. Therefore, additional patient consent was not required.

## Results

Study population

A total of 100 individuals with confirmed HIV infection were included in the study. Neurological manifestations were present in 44 (44%) patients, while 56 (56%) patients had no neurological involvement. Among patients with neurological manifestations, 27 (61.4%) had opportunistic CNS infections, most commonly tubercular meningitis (n = 16), followed by cryptococcal meningitis (n = 7) and toxoplasmosis (n = 4). The remaining 17 (38.6%) patients had non-infectious neurological diagnoses, including intracranial haemorrhage. At presentation, 18 (40.9%) neurological patients were receiving ART, while 26 (59.1%) were not on treatment.

Baseline characteristics

Baseline laboratory characteristics of the study population are summarised in Table 1. The mean CD4 count was comparable between patients with and without neurological manifestations (100.27 ± 48.20 vs. 102.00 ± 44.28 cells/µL). Haemoglobin levels were also similar between the two groups (9.57 ± 2.29 vs. 9.76 ± 2.10 g/dL). No statistically significant differences were observed in total leucocyte count, serum creatinine, or serum sodium levels between patients with and without neurological involvement. Liver function parameters, including AST, ALT, and bilirubin, were similar between patients with and without neurological manifestations.

**Table 1:** Baseline laboratory characteristics of the study population. Values are presented as mean ± SD or median (IQR) for continuous variables. CD4 count is expressed as cells/µL. Haemoglobin is expressed in g/dL. Serum creatinine is expressed in mg/dL. Serum sodium is expressed in mmol/L. AST and ALT are expressed in U/L, and bilirubin in mg/dL. AST = aspartate aminotransferase; ALT = alanine aminotransferase; SD = standard deviation; IQR = interquartile range

Parameter	Neurological (n = 44), mean ± SD	Neurological, median (IQR)	Non-neurological (n = 56), mean ± SD	Non-neurological, median (IQR)
CD4 (cells/µL)	100.27 ± 48.20	88.5 (76.0–128.0)	102.00 ± 44.28	98.0 (78.0–128.5)
Haemoglobin (g/dL)	9.57 ± 2.29	9.0 (7.95–10.85)	9.76 ± 2.10	9.4 (8.0–10.85)
Total leukocyte count (cells/µL)	5,081.82 ± 2,270.64	4,650 (3,424–6,050)	5,150.79 ± 2,346.42	4,700 (3,424–6,267)
Creatinine (mg/dL)	1.08 ± 0.58	0.95 (0.68–1.42)	1.14 ± 0.59	0.95 (0.80–1.65)
Sodium (mmol/L)	134.16 ± 6.33	135.0 (130.0–139.25	134.45 ± 6.44	135.5 (130.75–139.25)
Total bilirubin (mg/dL)	0.81 ± 0.41	0.8 (0.5–1.03)	0.87 ± 0.38	0.85 (0.5–1.2)
AST (U/L)	45.93 ± 21.16	40.5 (30–52)	49.18 ± 19.60	43 (36.25–60.5)
ALT (U/L)	41.82 ± 16.86	36 (31.75–49)	43.02 ± 14.99	37 (33–49)

Distribution of neurological diagnoses

Among patients presenting with neurological manifestations (n = 44), opportunistic CNS infections were the predominant aetiology, accounting for 27 (61.4%) cases (Table 2). Tubercular meningitis was the most frequent diagnosis, observed in 16 (36.4%) patients, followed by cryptococcal meningitis in 7 (15.9%) patients and toxoplasmosis in 4 (9.1%) patients. Non-opportunistic (non-infectious) neurological diagnoses comprised 17 (38.6%) cases, including cerebrovascular events such as intracranial haemorrhage. Overall, infectious causes represented the majority of neurological presentations in this cohort.

**Table 2:** Distribution of neurological diagnoses among patients with neurological manifestations (n = 44). Values are presented as number (percentage). Opportunistic infections include tubercular meningitis, cryptococcal meningitis, and toxoplasmosis. Non-opportunistic diagnoses include non-infectious neurological conditions such as cerebrovascular events (e.g. intracranial haemorrhage).

Diagnosis category	n (%)
Tubercular meningitis	16 (36.4%)
Cryptococcal meningitis	7 (15.9%)
Toxoplasmosis	4 (9.1%)
Non-opportunistic neurological diagnoses (including haemorrhage)	17 (38.6%)

Multivariable analysis of predictors of neurological manifestations

A multivariable logistic regression analysis was performed to identify independent predictors of neurological manifestations in the overall cohort (n = 100). Variables included in the model were CD4 count, haemoglobin, total leucocyte count, serum creatinine, sodium, liver function parameters (AST/ALT and total bilirubin), age, sex, and ART status. None of the evaluated variables demonstrated a statistically significant independent association with neurological manifestations at presentation (all p > 0.05) (Table 3). These findings suggest that routinely available laboratory parameters and baseline clinical characteristics may have limited discriminatory value in predicting neurological involvement in this cohort.

**Table 3:** Multivariable logistic regression analysis for predictors of neurological manifestations (overall cohort, n = 100). Values represent adjusted associations derived from multivariable logistic regression analysis. Variables included in the model were CD4 count, haemoglobin, total leucocyte count, serum creatinine, sodium, liver function parameters (AST/ALT and total bilirubin), age, sex, and ART status. Statistical significance was defined as p-values <0.05. AST = aspartate aminotransferase; ALT = alanine aminotransferase; ART = antiretrovial therapy; OR = odds ratio; CI = confidence interval

Variable	Adjusted OR	95% CI	P-value
CD4 (per 50-cell decrease)	1.00	0.63–3.32	0.383
ART status	1.45	0.63–2.86	0.742
Haemoglobin	0.97	0.79–1.18	0.747
Total leukocyte count	1.00	1.00–1.00	0.998
Creatinine	0.79	0.38–1.65	0.537
Sodium	0.98	0.92–1.05	0.593
Total bilirubin	0.67	0.22–2.06	0.486
AST	0.99	0.96–1.02	0.502
ALT	1.00	0.96–1.04	0.848
Age	1.00	0.95–1.04	0.876
Sex	1.16	0.47–2.86	0.496

Comparison between patients with and without neurological manifestations

There was no significant difference in the mean CD4 count between patients with and without neurological involvement (100.27 ± 48.20 vs. 102.00 ± 44.28 cells/µL; p = 0.85). Similarly, haemoglobin, total leukocyte count, creatinine, sodium, and liver function parameters demonstrated no statistically significant between-group differences (all p > 0.05).

Threshold analysis of CD4 count

To further assess the relationship between immune status and neurological involvement, threshold analyses were performed using commonly applied CD4 cut-offs (<50, <100, and <150 cells/µL) (Table 4). No significant association was observed between CD4 strata and neurological manifestations. The distribution of patients across these categories remained comparable between the two groups.

**Table 4:** Association between CD4 count threshold and neurological manifestations in patients with HIV. Values are presented as number (percentage). P-values were calculated using Fisher’s exact test. Each CD4 threshold represents a cumulative subset of the cohort, with lower thresholds nested within higher thresholds, rather than mutually exclusive groups.

CD4 threshold (cells/µL)	Neurological manifestations, n (%)	No neurological manifestations, n (%)	Total (n)	P-value
<50	6 (40%)	9 (60%)	15	0.785
<100	26 (46.4%)	30 (53.6%)	56	0.686
<150	39 (43.8%)	50 (56.2%)	89	1.00

Multivariable analysis of predictors of infectious neurological aetiology

Within the neurological cohort (n = 44), multivariable logistic regression analysis identified lower CD4 count as an independent predictor of infectious neurological aetiology. Specifically, for every 50-cell decrease in CD4 count, the odds of an infectious diagnosis increased significantly (adjusted OR = 4.30, 95% CI = 1.18-15.63; p = 0.027) (Table 5). No significant independent associations were observed for haemoglobin, total leucocyte count, serum creatinine, sodium, liver function parameters, age, sex, or ART status (all p > 0.05).

**Table 5:** Multivariable logistic regression analysis for predictors of infectious neurological aetiology in patients with neurological manifestations (n = 44). Values represent adjusted ORs with 95% cCIs derived from multivariable logistic regression analysis. CD4 count was modelled per 50-cell decrease. Statistical significance was defined as p-values <0.05. AST = aspartate aminotransferase; ALT = alanine aminotransferase; ART = antiretrovial therapy; OR = odds ratio; CI = confidence interval; NS = not significant

Variable	Adjusted OR	95% CI	P-value
CD4 (per 50-cell decrease)	4.30	1.18–15.63	0.027
ART status	NS	-	>0.05
Haemoglobin	NS	-	>0.05
Total leukocyte count	NS	-	>0.05
Creatinine	NS	-	>0.05
Sodium	NS	-	>0.05
Total bilirubin/AST/ALT	NS	-	>0.05
Age	NS	-	>0.05
Sex	NS	-	>0.05

Receiver operating characteristic curve analysis

**Figure 2:**
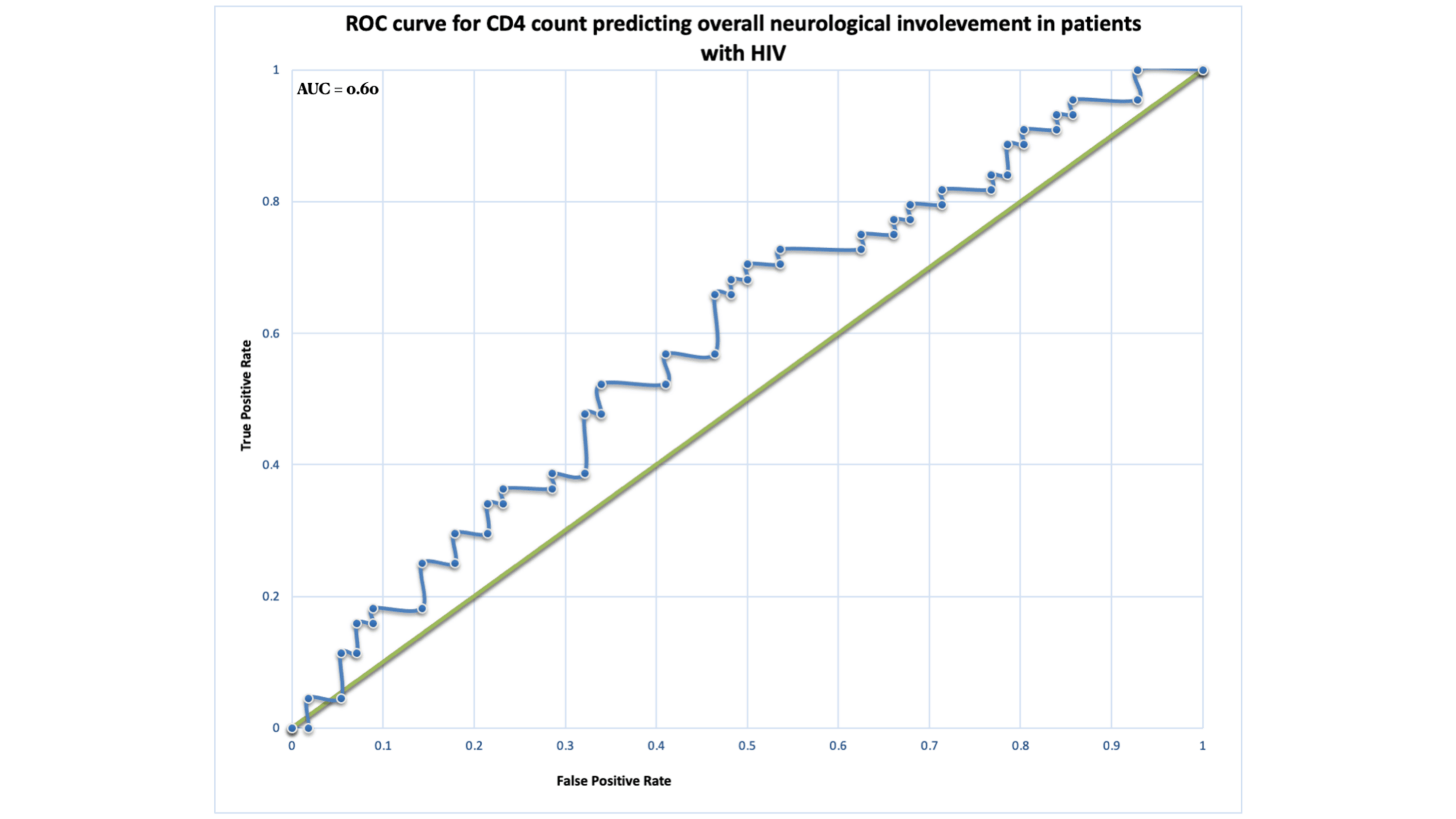
ROC curve for CD4 count predicting neurological involvement in HIV patients. ROC curve for CD4 count predicting neurological involvement in HIV patients. The model demonstrated poor discriminatory ability, with an AUC of 0.60, indicating limited utility of CD4 count alone in predicting overall neurological manifestations. The curved line represents the performance of CD4 count across different thresholds, while the diagonal reference line indicates no discrimination. ROC = receiver operating characteristic; AUC = area under the curve

**Figure 3:**
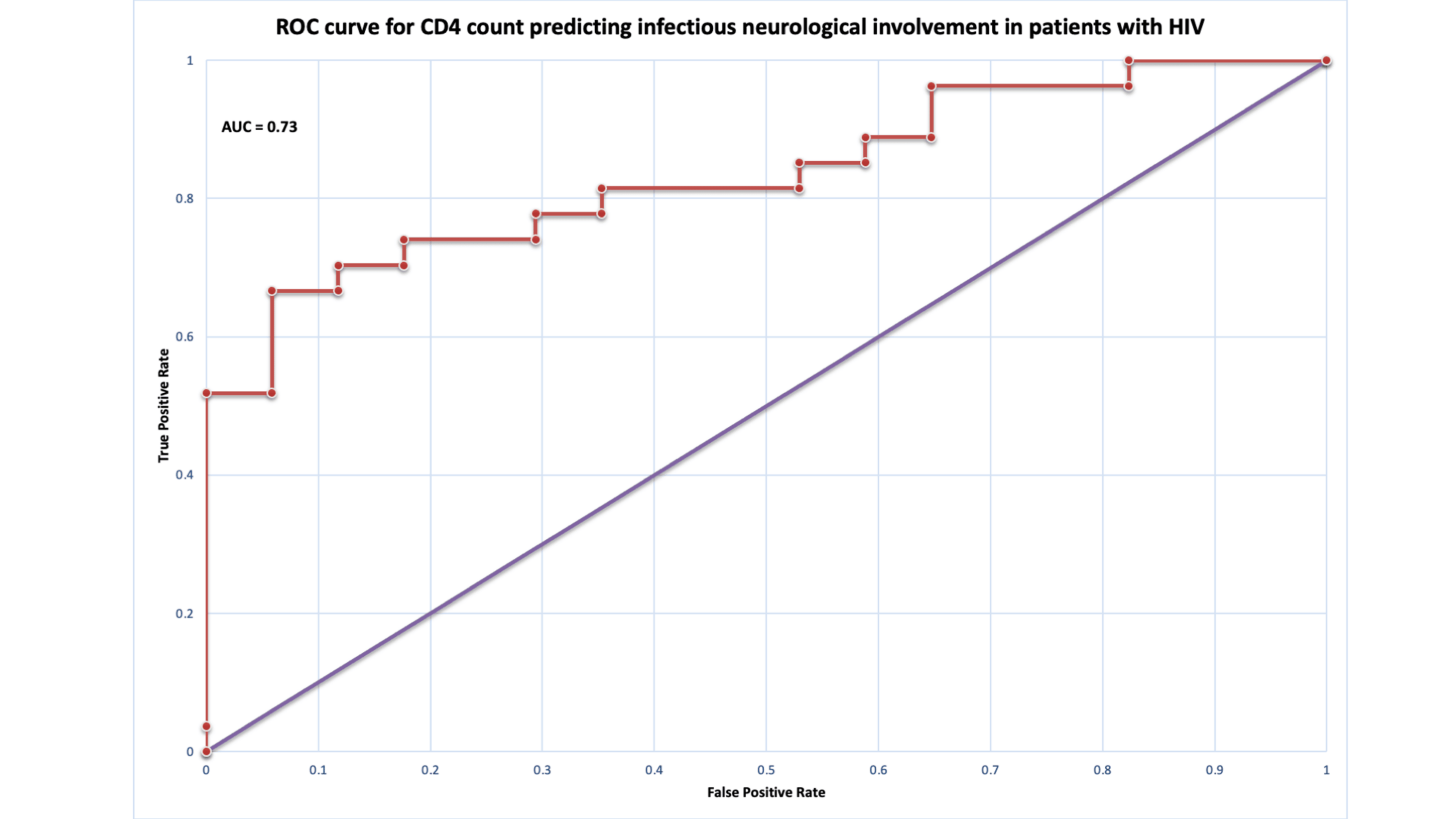
ROC curve for CD4 count predicting infectious neurological involvement in patients with HIV. The ROC curve illustrates the diagnostic performance of CD4 count in predicting infectious neurological involvement in patients with HIV. The ROC curved (solid line) demonstrates moderate discrimination, with an AUC of 0.73. The diagonal reference line indicates no discriminatory ability. ROC = receiver operating characteristic; AUC = area under the curve

ROC curve analysis was performed to evaluate the discriminative ability of CD4 count in predicting neurological involvement and infectious neurological aetiology. In the overall cohort, CD4 count demonstrated poor discriminative ability for identifying neurological manifestations (AUC = 0.60) (Figure 2; Table 6), indicating performance close to chance. In contrast, within the neurological cohort, CD4 showed good discriminative ability for distinguishing infectious from non-infectious aetiologies (AUC = 0.73) (Figure 3).

**Table 6:** ROC curve analysis of CD4 count for prediction of neurological involvement and infectious aetiology. ROC analysis was performed to assess the discriminative performance of CD4 count. An AUC of 0.5 indicates no discrimination, 0.6-0.7 poor, 0.7-0.8 moderate, and >0.8 good discrimination. ROC = receiver operating characteristic; AUC = area under the curve

Model	AUC	Interpretation
Neurological prediction (overall cohort)	0.60	Poor discrimination (close to chance)
Infectious aetiology model (neurological cohort)	0.73	Moderate discrimination

Summary of findings

Overall, CD4 count and routinely available laboratory parameters did not demonstrate meaningful discriminatory value in identifying patients with neurological manifestations in the overall cohort. However, among patients presenting with neurological involvement, a lower CD4 count was independently associated with an infectious aetiology and showed moderate discrimination ability. In contrast, other routine laboratory parameters did not provide additional predictive value.

## Discussion

This secondary analytic study draws on a prospectively collected cohort study of adults living with HIV who were managed at a tertiary care centre between 2016 and 2017 [12], originally conducted as part of an MD thesis. While the initial dataset described the spectrum and prevalence of neurological manifestations, the present work focuses on whether CD4 count and routinely available laboratory parameters can help stratify neurological involvement at presentation and distinguish infectious from non-infectious aetiologies. This approach provides a clinically relevant assessment of the practical utility and limitations of CD4 count in routine HIV care.

Neurological complications continue to represent a major contributor to morbidity and mortality among people living with HIV worldwide. In particular, Marais et al. and Thwaites et al. have underscored the severe neurological outcomes and high mortality associated with HIV-related tuberculous meningitis, particularly in individuals with advanced immunosuppression [13,14]. In parallel, Rajasingham et al. showed that cryptococcal meningitis continues to account for a substantial proportion of HIV-related deaths globally, with a disproportionate burden among patients with CD4 counts below 100 cells/µL [15]. Despite wider access to ART, subsequent work has confirmed that this burden persists, especially in resource-limited settings [16].

These patterns are mirrored in our cohort, where opportunistic CNS infections made up the majority of neurological presentations. However, an important nuance emerges from our analysis. Although CD4 count has long been used as a surrogate marker of immunological vulnerability, it did not independently predict the presence of neurological manifestations in the overall cohort once we adjusted for other variables. The discriminatory performance of CD4 alone in the overall cohort was modest (AUC = 0.60), suggesting that relying solely on CD4 count to identify patients at risk of neurological involvement at presentation is likely to be inadequate.

This finding stands in contrast to earlier cross-sectional studies, particularly from low- and middle-income settings, where lower CD4 counts were frequently associated with a higher burden of neurological disease. For instance, Sharma et al. reported increased frequencies of CNS infections among patients with lower CD4 strata [2], while Dian et al. demonstrated a strong association between advanced immunosuppression and opportunistic CNS infections such as cerebral toxoplasmosis in HIV cohorts [17]. However, such studies were largely based on unadjusted analyses or focused predominantly on infectious aetiologies. In contrast, our approach incorporated a broad clinical context, including haematological, renal, serum sodium, hepatic, and ART-related variables, thereby accounting for systemic confounders that may influence neurological presentation. The attenuation of CD4 significance in predicting “any neurological involvement” in our study likely reflects the evolving and heterogeneous nature of neurological disease in the contemporary HIV population, where both infectious and non-infectious mechanisms contribute to clinical presentation.

The lack of clear association between CD4 count and overall neurological involvement in our cohort likely reflects the changing epidemiology of HIV in the ART era. Phiri et al. highlighted the increasing role of cerebrovascular disease and vascular mechanisms in contributing to neurological morbidity among treated HIV populations [18]. In addition, Heaton et al. described persistent neurocognitive impairment driven by inflammatory processes, often seen across a wider range of CD4 counts [19]. Taken together, these observations suggest that neurological complications in contemporary HIV are increasingly multifactorial and do not follow a simple linear relationship with CD4 levels alone, which may explain the limited discriminative ability observed in our overall cohort.

In contrast to the overall cohort, a more consistent pattern emerged when the analysis was restricted to patients with neurological involvement. Within this subgroup, CD4 count showed a clearer association with infectious aetiologies, with moderate discriminatory ability (AUC = 0.73). This improvement is not unexpected. In the broader cohort, neurological presentations are heterogeneous, whereas infectious CNS conditions tend to follow a more direct relationship with the degree of immunosuppression. This observation is consistent with evidence that advanced CD4 depletion remains a key determinant of opportunistic CNS infections. Clinical studies have shown that patients with lower CD4 count are significantly more likely to develop infections such as cryptococcal meningitis, cerebral toxoplasmosis, and tuberculous meningitis, particularly below the defined immunological threshold [20-22]. In this context, CD4 count appears to retain clinical relevance, not as a universal predictor of neurological disease, but as a marker that helps distinguish infectious from non-infectious aetiologies once neurological involvement is established.

Beyond immunological markers, routinely available laboratory parameters did not demonstrate independent predictive value in our adjusted analysis. Haematological indices, particularly anaemia, were not associated with neurological involvement after multivariable adjustment. Although anaemia remains highly prevalent among people living with HIV and has consistently been linked to increased mortality, it more likely reflects overall systemic disease burden than CNS-specific pathology [8,9]. Our findings therefore suggest that anaemia should be interpreted as a marker of advanced illness rather than a direct predictor of neurological involvement.

A similar pattern was observed with renal function. Serum creatinine did not independently predict neurological manifestations in our cohort. Chronic kidney disease is increasingly recognised among HIV-infected individuals receiving ART, but it is more plausibly a reflection of cumulative comorbidity, treatment exposure, and systemic illness than a direct driver of neurological disease [10].

Likewise, standard hepatic parameters (bilirubin, AST, ALT) were not independently associated with neurological involvement in this cohort, suggesting they more likely indexed overall systemic illness than brain-specific pathology. Liver dysfunction is frequent among people living with HIV due to co-infections and antiretroviral-related hepatotoxicity [11], but current data do not support a direct mechanistic link between isolated liver test abnormalities and CNS presentation, aligning our findings with this broader literature.

Electrolyte derangements such as hyponatraemia are well described in CNS infections, including tuberculous meningitis, where they correlate with illness severity rather than providing disease-specific prognostic information. In keeping with this, serum sodium did not independently discriminate neurological involvement in our analysis, reinforcing the interpretation of these abnormalities as markers of systemic decompensation rather than targeted neurological risk [23].

ART exposure similarly failed to emerge as an independent predictor of neurological manifestations. This likely reflects the complex interplay between immune restoration and inflammatory responses. Clinical trials have demonstrated that the timing of ART initiation in CNS infections, particularly tuberculous and cryptococcal meningitis, can significantly influence outcomes, sometimes worsening inflammatory complications when initiated early [24,25]. These findings highlight the nuanced role of immune reconstitution and suggest that ART status alone lacks sufficient specificity to function as a predictive variable.

Finally, the absence of viral load data represents a limitation but also reflects real-world constraints in mainly low- and middle-income settings. While viral load measurement remains central to monitoring treatment response and disease progression, its role in predicting neurological manifestations is less clearly defined and likely influenced by multiple interacting factors [26]. The model discrimination performance observed in our analysis reinforces an important methodological consideration: conventional regression models assume linear relationships, whereas HIV-associated neurological diseases arise from complex, non-linear interactions involving immune suppression, inflammation, co-infections, vascular mechanisms, and host susceptibility. Recent methodological literature suggests that machine learning approaches may offer advantages over traditional regression models when dealing with complex, high-dimensional clinical data. However, their performance remains variable and is closely dependent on study design, data quality, and appropriate validation [27,28]. Rather than consistently outperforming conventional models, current evidence emphasises the need for cautious interpretation when applying these techniques in clinical prediction settings.

While our findings provide insight into the potential role of CD4 count and routinely available laboratory parameters in HIV-related neurological disease, their clinical interpretation requires careful consideration. The overall discriminatory performance observed in our analysis was modest, with an AUC of approximately 0.60, indicating limited ability to distinguish outcomes beyond chance. This suggests that, despite observed statistical associations, these parameters alone are unlikely to serve as reliable standalone tools for clinical decision-making. These findings should be interpreted in the context of important methodological and clinical constraints. The relatively small sample size, particularly within subgroup analyses, may have limited model stability and reduced the ability to detect subtle but clinically meaningful associations.

In addition, as a secondary analysis of an existing dataset, the study is subject to inherent limitations, including potential variability in diagnostic classification and limited control over data collection processes. The absence of key variables, such as viral load, further restricts the biological interpretation of these findings, given its central role in HIV disease activity. The data were derived from a cohort studied between 2016 and 2017, and although core immunological relationships are unlikely to have fundamentally changed, advances in ART and evolving treatment paradigms may limit direct applicability to contemporary clinical practice. Furthermore, the single-centre, tertiary care setting introduces the possibility of selection bias, with a higher likelihood of more severe or complex cases being represented.

Importantly, while CD4 count demonstrated some association within the neurological subgroup, routinely available laboratory parameters did not show consistent discriminatory value. This reinforces the likelihood that neurological manifestations in HIV are driven by complex, multifactorial processes that cannot be adequately captured using isolated clinical or biochemical markers. Taken together, these findings should be interpreted as exploratory and hypothesis-generating rather than definitive evidence for clinical application. Future studies incorporating larger datasets and more comprehensive biological markers will be essential to better characterise these relationships.

Strengths

A key strength of the study lies in its detailed clinical phenotyping, with diagnoses supported by imaging and CSF analysis wherever applicable. This reduces diagnostic uncertainty and allows for a more reliable classification of neurological involvement, which is particularly important in heterogeneous conditions such as HIV-associated CNS disease. In addition, the study adopts a multivariable analytic approach incorporating routinely available laboratory parameters, including haematological, renal, and hepatic indices. This reflects real-world clinical practice, where decisions are often based on accessible investigations rather than specialised biomarkers. By evaluating these variables together, the analysis provides a more clinically meaningful assessment of their independent predictive value.

Another strength is the use of ROC-based discrimination analysis. Rather than relying solely on statistical associations, the study specifically evaluates how well these parameters perform in distinguishing patients with and without neurological involvement. This focus on predictive performance enhances the practical interpretation of the findings and alliance with contemporary approaches to clinical risk stratification. Finally, data from low- and middle-income settings examining neurological manifestations of HIV using structured predictive approaches remain limited. This study, therefore, contributes valuable context-specific evidence, particularly in environments where resource constraints necessitate reliance on simple, scalable clinical tools.

Limitations

Several limitations should be considered when interpreting these findings. The single-centre design may limit generalisability, as patient characteristics, disease patterns, and healthcare access can vary across regions and settings. As a secondary analysis, the study was inherently constrained by the variables available in the original dataset. Important parameters such as viral load and detailed ART regimen stratification were not included, which may have limited a more comprehensive assessment of disease dynamics and treatment effects.

In addition, detailed information regarding laboratory assay platforms and calibration methods was not available within the original dataset, which may introduce minor variability in measured parameters. Furthermore, as a secondary analysis, certain granular details regarding patient recruitment and the screening process were not systematically recorded, which may limit the reproducibility of the analysis.

The data were derived from a cohort studied between 2016 and 2017. Although this may not fully reflect contemporary ART practices and evolving epidemiological trends, the core biological relationships between immunosuppression and neurological disease are unlikely to have fundamentally changed. Moreover, such data remain relevant to many resource-limited settings where similar clinical challenges persist.

The sample size, while adequate for exploratory modelling, may have limited the detection of smaller associations or more complex interactions between variables. In addition, the observational design precludes causal inference, and residual confounding cannot be entirely excluded despite multivariable adjustment. Further, given the sample size relative to the number of variables included, there is a possibility of model overfitting, a type II error, and the findings should be interpreted with appropriate caution.

Future directions

The findings from this study point towards several important next steps. First, prospective validation in larger, multicentre cohorts is needed to confirm whether the patterns observed here hold across different populations and healthcare settings. Such studies should incorporate richer clinical datasets, including viral load measurements, detailed antiretroviral regimen information, and longitudinal follow-up, to better capture the dynamic interplay between treatment, immune recovery, and neurological risk. Second, there is potential for integrating multidimensional clinical and laboratory data into composite risk-assessment frameworks. Given the non-linear, multifactorial nature of HIV-associated neurological disease, approaches that move beyond single-parameter thresholds, such as composite clinical scores, may improve risk stratification, particularly in settings where early intervention could meaningfully alter outcomes. Such tools must be developed transparently, tailored to local epidemiological contexts, and rigorously validated before clinical implementation. Finally, future work should prioritise the development of scalable, practical risk-assessment frameworks designed specifically for resource-limited environments. These tools need to balance predictive performance with feasibility, leveraging routinely available data without requiring infrastructure that remains out of reach in many low- and middle-income settings. Only through this pragmatic lens can predictive research translate into real-world clinical benefit where it is needed most.

## Conclusions

In this study, CD4 count was not an independent predictor of overall neurological involvement in adults living with HIV, although it remained significantly associated with infectious neurological aetiologies. These findings suggest that while CD4 count reflects the degree of immunosuppression, it has limited utility as a standalone marker for assessing overall neurological risk. From a clinical perspective, CD4 count should, therefore, not be used in isolation, but rather interpreted within the broader clinical context. Routine laboratory parameters similarly demonstrated limited discriminating value, reinforcing the multifactorial and complex nature of HIV-associated neurological disease. Overall, our findings suggest that no single marker is sufficient for risk stratification. These findings highlight the continued importance of comprehensive clinical assessment rather than reliance on isolated laboratory markers. Future approaches should focus on integrating clinical and laboratory data to improve early identification of high-risk patients, particularly in resource-limited settings where practical and scalable tools are essential. Taken together, our findings support a more comprehensive clinical approach to risk assessment, where laboratory markers are considered alongside clinical presentation rather than relied upon independently. Importantly, these results should be interpreted cautiously given the study’s observation design, relatively small sample size, and potential for residual confounding. Accordingly, the findings are best viewed as exploratory and hypothesis-generating, and questions are warranted when generalising beyond similar clinical settings.
